# Effective Healing of *Staphylococcus aureus*-Infected Wounds in Pig Cathelicidin Protegrin-1-Overexpressing Transgenic Mice

**DOI:** 10.3390/ijms241411658

**Published:** 2023-07-19

**Authors:** Nagasundarapandian Soundrarajan, Prathap Somasundaram, Dohun Kim, Hye-Sun Cho, Hyoim Jeon, Byeonyong Ahn, Mingue Kang, Hyuk Song, Chankyu Park

**Affiliations:** Department of Stem Cells and Regenerative Biology, Konkuk University, Hwayang-dong, Seoul 05029, Republic of Korea; sundarmeets@gmail.com (N.S.); prathaparunms@gmail.com (P.S.); kdh2136@konkuk.ac.kr (D.K.); chssky77@gmail.com (H.-S.C.); kamuijhi@naver.com (H.J.); anhbyeongyong1123@gmail.com (B.A.); mingue5349@gmail.com (M.K.); songh@konkuk.ac.kr (H.S.)

**Keywords:** wound healing, antimicrobial peptides, cathelicidin, protegrin-1, cell migration

## Abstract

Antimicrobial peptides (AMPs) are promising alternatives to existing treatments for multidrug-resistant bacteria-infected wounds. Therefore, the effect of protegrin-1 (PG1), a potent porcine AMP with broad-spectrum activity, on wound healing was evaluated. PG1-overexpressing transgenic mice were used as an in vivo model to evaluate its healing efficiency against *Staphylococcus aureus*-infected (10^6^ colony forming units) wounds. We analyzed the wounds under four specific conditions in the presence or absence of antibiotic treatment. We observed the resolution of bacterial infection and formation of neo-epithelium in *S. aureus*-infected wounds of the mice, even without antibiotic treatment, whereas all wild-type mice with bacterial infection died within 8 to 10 days due to uncontrolled bacterial proliferation. Interestingly, the wound area on day 7 was smaller (*p <* 0.01) in PG1 transgenic mice than that in the other groups, including antibiotic-treated mice, suggesting that PG1 exerts biological effects other than bactericidal effect. Additionally, we observed that the treatment of primary epidermal keratinocytes with recombinant PG1 enhanced cell migration in in vitro scratch and cell migration assays. This study contributes to the understanding of broad-spectrum endogenous cathelicidins with potent antimicrobial activities, such as PG1, on wound healing. Furthermore, our findings suggest that PG1 is a potent therapeutic candidate for wound healing.

## 1. Introduction

Skin prevents the infiltration of pathogens into the body [1]. Wound healing is an important process that maintains skin health and is mediated by various cellular components, including the skin matrix and immune cells [1,2,3]. This complex process takes place through four distinct phases: hemostasis, inflammation, proliferation, and remodeling. These phases are interconnected, and several components are involved in tissue repair [4,5,6]. However, infections of opportunistic microbes, such as *Staphylococcus*, *Pseudomonas*, and multidrug-resistant (MDR) microbes, can prolong the inflammatory phase [4,5,6]. Conventional antibiotics and drugs often fail to promote healing because of limited penetration through biofilms [6,7], poor activity against drug-resistant microbes, and failure to counteract bacterial toxins [8,9,10]. Therefore, chronic non-healing wounds have become a serious health issue.

Antimicrobial peptides (AMPs) are a component of host innate immunity that provide first-line defense against invading pathogens [11,12]. These molecules exhibit broad-spectrum antimicrobial activity and are also involved in immunomodulation, cell proliferation, and the neutralization of pro-inflammatory substances [13,14,15,16,17]. These properties make AMPs promising alternatives to conventional antibiotics, which is of specific importance owing to the emergence of MDR pathogens. In addition, AMPs accelerate wound healing by promoting angiogenesis and re-epithelization [13,18,19].

Cathelicidins are a family of AMPs with broad-spectrum antimicrobial activities that are relatively potent compared to other AMP families [20,21]. Although humans and mice each have a single cathelicidin gene (*CAMP*), multiple cathelicidin genes are present in the genomes of other mammalian species [22,23,24,25]. Cathelicidins play a crucial role in wound healing [13,26,27,28]. Mice deficient in *CAMP* are susceptible to bacterial skin and urinary tract infections [29]. A recent study showed that the inhibition of the expression of cathelicidin-related antimicrobial peptides in mice inhibited the healing of infected wounds [30]. Conversely, PR39 overexpression protected transgenic (Tg) mice against *streptococcal* skin infections [31]. Several in vitro studies that used topical treatment composed of AMPs have highlighted the beneficial effects of AMPs on wound healing [13,17,27]. However, a comparison of the apparent advantages of AMPs over conventional antibiotics against pathogenic infections in in vivo systemic environments is limited [32,33].

Protegrin-1 (PG1), a cathelicidin that was originally identified in pig neutrophils, exhibits a broad range of antimicrobial activities [24,34,35]. PG1 can eliminate biofilms and prevent biofilm formation and therefore plays a role in wound healing in methicillin-resistant *Staphylococcus aureus*-infected tissues [36]. We recently reported that PG1 Tg mice infected with *S. aureus* exhibit bacterial clearance, reduced microscopic pulmonary injuries, and lower levels of pro-inflammatory cytokines than their wild-type counterparts [37]. Similarly, the ectopic expression of PG1 improved the survival of Tg mice following *Actinobacillus suis* infection [38]. The findings of these studies prompted us to evaluate the efficacy of PG1 against bacterial infection in wound healing in PG1 Tg mice. In the present study, we demonstrated the successful elimination of *S. aureus* infection and the healing of experimental wounds in PG1 Tg mice. Our findings provide additional evidence supporting the use of cathelicidins as topical therapeutic agents against bacterial skin infections.

## 2. Results and Discussion

### 2.1. Enhanced Wound Healing in Staphylococcus aureus-Infected Wounds in PG1 Tg Mice

**Figure 1:**
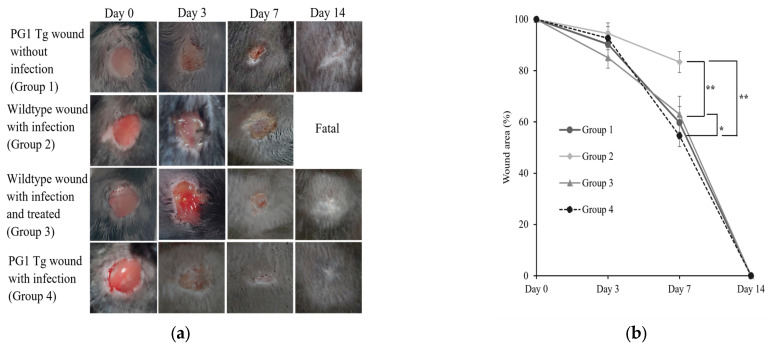
Wound healing in protegrin-1 (PG1)-overexpressing transgenic and wild-type mice after *Staphylococcus aureus* infection. (**a**) Representative images of wound healing at different time points after wound creation on the back of the mice and infection with *S. aureus* in the different treatment groups. The treatment groups and days of wound healing are indicated on the left and top, respectively. Group 1, PG1 transgenic mice with uninfected wounds; Group 2, wild-type mice with *S. aureus*-infected wounds; Group 3, wild-type mice with *S. aureus*-infected wounds that were treated with a single-dose of gentamycin every 24 h for three consecutive days; and Group 4, PG1 transgenic mice with *S. aureus*-infected wounds. “Fatal” indicates that no mice survived. (**b**) The diameters of the unclosed wound areas were measured on days 0, 3, 7, and 14, and the wound area percentage compared to the wound size on day 0 was calculated. Statistical significance of the differences in wound size between pairwise comparisons of each different treatment group is shown: * Group 3 vs. 4, *p <* 0.05; ** Group 4 vs. 2 and Group 3 vs. 2, *p <* 0.01, respectively. PG1, protegrin-1; Tg, transgenic.

Sterile wounds were generated on the backs of wild-type and PG1 Tg mice, i.e., which express low levels of PG1 in the skin [37]. In the bacterial infection group, wounds were infected with 10^6^ colony-forming units (CFUs) of *S. aureus* ATCC 6538. In the antibiotic treatment group, a single-dose gentamycin (2 μg/mL) solution was applied to the wounds every 24 h for three consecutive days. The resultant four experimental groups (*n* = 5/group) are as follows: Group 1, PG1 Tg mice with uninfected wounds; Group 2, wild-type mice with *S. aureus*-infected wounds; Group 3: wild-type mice with *S. aureus*-infected wounds administered to gentamycin treatment; and Group 4, PG1 Tg mice with *S. aureus*-infected wounds. Animal health and wounds were monitored every 12 h until the last day of the experiment (day 14). All animals in Groups 1, 2, and 4 fully recovered, with re-epithelialization on day 14. In contrast, all animals in Group 2 died between days 8 and 10 (Figure 1a). Upon visual examination, heavier pus formation was observed in the Group 2 animals than in those in the other groups, which had relatively clean wounds. Although we did not perform postmortem pathological analysis, the death of Group 2 animals was likely caused by the uncontrolled proliferation of bacteria, leading to associated inflammatory responses and sepsis.

The sizes of the wounds were measured on days 0, 3, 7, and 14 (Figure 1b). Interestingly, PG1 Tg mice (Group 4) exhibited relatively fast wound closure on day 7 compared to that in the wild-type-infected group (Group 2, *p <* 0.01). We also found that PG1 Tg mice exhibited better wound closure than the antibiotic-treated mice (Group 3) (*p <* 0.05), suggesting that wound closure in PG1 Tg mice may be accelerated, in part, via enhanced proliferation and migration of epithelial cells by ectopically expressed PG1. We previously reported that in vitro treatment with ΔPbCATH04, a python cathelicidin, effectively healed *S. aureus*-infected wounds by eliminating the pathogen and promoting wound closure [13]. These results indicate that cathelicidins with potent antimicrobial activities can be effective in healing cutaneous bacteria-infected wounds.

### 2.2. Bacterial Clearance in Infected Wounds of PG1 Tg Mice

To determine the in vivo effect of PG1 on the clearance of bacteria from infected cutaneous wounds of PG1 Tg mice, we quantified the bacterial load in wound biopsy specimens of each experimental group. The total CFUs were estimated from the tissue lysates of day 0, 3, 7, and 14 wound samples (Table 1). Group 1 showed negligible bacterial counts across all analyzed time points. Group 2 showed a 100-fold increase in the CFU count on day 3 (8.21 ± 3.4 × 10^8^ CFUs/g) and day 7 (8.75 ± 3.4 × 10^8^ CFUs/g) compared to that on day 0. In contrast, the CFU counts decreased 10 and 100-fold in the day 3 samples of Groups 3 and 4, respectively, compared to that in the day 0 samples. In the day 7 samples of Groups 3 and 4, the CFU counts decreased further to 1.41 ± 0.9 × 10^2^ and 1.39 ± 2.14 × 10^2^, respectively, and the counts were almost negligible in the day 14 samples, similar to that of Group 1, which had no bacterial infection. Interestingly, Group 4 showed a 10-fold lower CFU count than the gentamycin-treated wounds (Group 3) on days 3 and 14 (Table 1 and Figure S1), suggesting a slightly better bacterial clearance in the wounds of PG1 Tg mice than that observed with antibiotic treatment in wild-type mice. The synergistic effects of ectopically expressed PG1 and other endogenously expressed antimicrobial molecules in wounds may exert synergistic effects on bacterial clearance. The immunomodulatory effects of PG1 may also contribute to bacterial clearance in wounds of PG1 Tg mice. Bacterial clearance and wound healing in the skin of PG1 Tg mice were consistent with the results of our previous study, in which PG1 Tg mice challenged with *S. aureus* showed bacterial clearance equivalent to that of conventional antibiotics as well as improved survival [37].

### 2.3. Efficient Formation of Neo-epithelium in PG1 Tg Mice with Infected Wounds 

**Figure 2:**
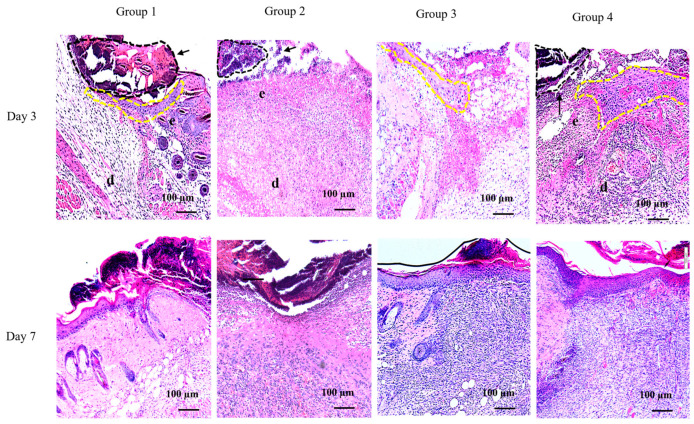
Histological analysis of wound sections of PG1-overexpressing transgenic and wild-type mice after *Staphylococcus aureus* infection. Hematoxylin and eosin-stained representative sections of wounds infected with *S. aureus* excised on days 3 and 7 (20× magnification). Letters in the image correspond to: e, epidermis; and d, dermis. The formation of neo-epithelium (yellow dotted lines) and dead tissue eschar (black dotted lines) indicated by black arrow. Group 1, PG1 transgenic mice with uninfected wounds; Group 2, wild-type mice with *S. aureus*-infected wounds; Group 3, wild-type mice with *S. aureus*-infected wounds that were treated with a single-dose of gentamycin every 24 h for three consecutive days; and Group 4, PG1 transgenic mice with *S. aureus*-infected wounds. The formation of neo-epithelium layer was pronounced in Groups 1, 3, and 4, compared to Group 2 in both day 3 and 7 sections. The re-establishment of epidermal structure is more evident on day 3 than on day 7.

We assessed the wound biopsy sections of post-wound days 3 and 7 samples using hematoxylin and eosin (H&E) staining (Figure 2). Although experimental wound creation was carefully conducted, eschar on the reforming epithelium and remaining fragile tissues at the wound area prevented a clean morphology of the tissue sections. Histological observations showed that the reformation of the neo-epithelium was clearly identifiable in the sections of Groups 1, 3, and 4. However, this was not clear for Group 2. Representative sections of post-wound day 7 showed a more complete epithelial layer structure, distinguishing different sublayers of the epidermis, stratum corneum, stratum lucidum, and stratum granulosum in Groups 1, 3, and 4, but not in Group 2 (Figure 2). The degree of neo-epithelial layer reestablishment was similar in Groups 3 and 4 and was more complete than that in Group 1. The reformation of the neo-epithelial layer in Group 2 was less complete than that in the other experimental groups.

**Figure 3:**
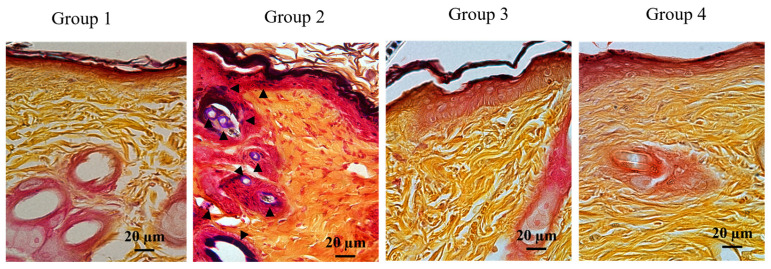
Histological evaluation of the resolution of bacterial infection in *Staphylococcus aureus*-infected wounds of PG1 transgenic mice using Gram staining. Representative Gram staining images (600×) of sections of day 14 infected wounds. Group 1, PG1 transgenic mice with uninfected wounds; Group 2, wild-type mice with *S. aureus*-infected wounds; Group 3, wild-type mice with *S. aureus*-infected wounds that were treated with a single-dose of gentamycin every 24 h for three consecutive days; and Group 4, PG1 transgenic mice with *S. aureus*-infected wounds. For Group 2, day 7 samples were used for Gram staining because all animals died before day 14. Gram-positive stains in purple blue are indicated with black arrowheads in various regions of the epidermis in Group 2. However, no positive signals were discernable in the epidermis of day 14 samples of Groups 1, 3, and 4.

To assess clearance of bacteria in the neo-epithelium, Gram staining was performed on sections of tissues after day 14 of wounding, i.e., when the wounds were completely closed (Figure 3). Because none of the Group 2-animals past day 10, sections of post-wound day 7 tissues were analyzed. Gram-positive signals were not observed in the sections of Groups 1, 3, and 4, indicating bacterial clearance in the infected area. In contrast, we observed multiple Gram-positive signals across the epidermis in the day 7 sections of Group 2, including around the hair follicles. These results are in clear agreement with the bacterial count (Table 1). Moreover, these findings further demonstrate that ectopically expressed PG1 contributes to bacterial clearance and regeneration of epithelium in infected wounds.

### 2.4. Successful Production of Recombinant PG1 Using a Bacterial Expression System

Recombinant PG1 was expressed as inclusion bodies consisting of aggregated inactive proteins from BL21 cells in 1 L of bacterial culture, using a green fluorescent protein-based AMP production system [39]. Ultimately, 13 mg of purified PG1 (>95% purity) (Figure S2) was obtained, and antibacterial activity was confirmed against *S. aureus* ATCC 6538 and *Escherichia coli* ATCC 25922 (Table S1). The minimum inhibitory concertation (MIC) against *S. aureus* and *E. coli* was 4 and 6 μg/mL, respectively, thus validating that the bactericidal activity of recombinant PG1 was similar to that reported previously [39].

### 2.5. Enhancement of Epidermal Keratinocyte Migration by PG1

**Figure 4:**
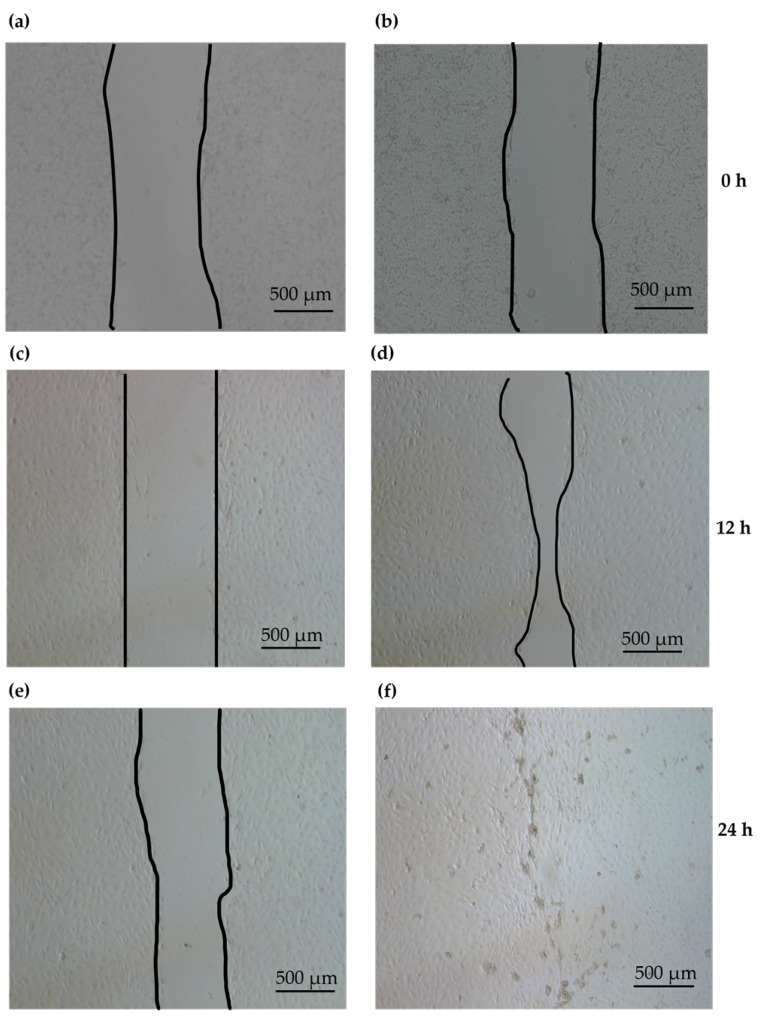
Effect of PG1 on cell scratch assay. A monolayer of cultured keratinocytes prepared from PG1 transgenic mice was scratched and treated with recombinant PG1. The culture time is indicated on the right. Panels (**a**,**c**,**e**) represent the untreated PG1 Tg keratinocytes, and (**b**,**d**,**f**) represent PG1 Tg keratinocytes treated with 8 μg/mL of PG1 at the indicated time points. Keratinocytes treated with PG1 exhibited wound repair activity in the scratched area. The edges of unfused scratch are indicated by black lines (**a**–**e**). Complete healing of the scratched area is shown in (**f**). Scale bar 500 μm.

Epidermal keratinocyte (KC) proliferation and migration are vital for re-epithelialization during wound healing [40]. AMPs can reportedly stimulate the proliferation and migration of KCs [17,27,41]. To evaluate the effect of PG1 on the migration capacity of KCs, primary KCs were isolated from the tail of PG1 Tg mice and subjected to a cell scratch assay. The effect of KC migration was evaluated after serum starvation. No wound repair activity was observed in the scratched area of the PG1 Tg KC monolayer, even after 24 h of culture (Figure 4a,c,e). We then evaluated the effect of recombinant PG1 (8 μg/mL) on KC migration. Interestingly, wound healing of PG1-treated Tg KCs was observed after 12 h of culture (Figure 4d), and the scratch was completely closed after 24 h (Figure 4f).

**Figure 5:**
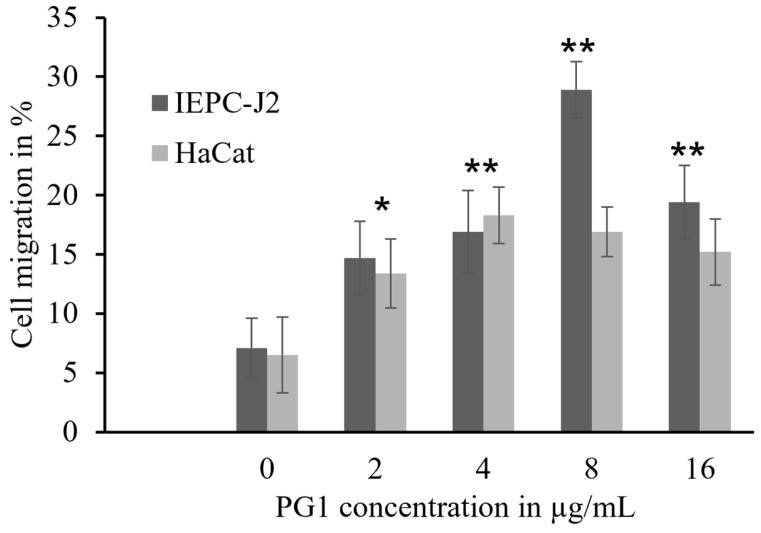
Effect of PG1 treatment on Transwell cell migration assay. Migration of PG1-treated IPEC-J2 and HaCaT cells was analyzed using the Transwell assay. PG1 (0–16 μg/mL) was added to the lower wells. Migration was assessed after 16 h of incubation, and the migrated cells were counted by Giemsa staining. Both cell lines exhibited a concentration-dependent increase in migration in response to PG1 treatment. All the experiments were repeated thrice, and the data is represented as the mean ± standard deviation. The statistical significance of migrated cell populations between the different treatment (PG1 concentrations 2–16 μg/mL) and untreated (PG1 concentration 0 μg/mL) groups was calculated by unpaired *t*-test; * *p <* 0.0005, ** p < 0.0001. PG1, protegrin-1.

KC primary cells exhibited >60% scratch repair after 12 h of PG1 treatment. This suggests that PG1 induces the migration of KCs to facilitate wound closure. However, the results also suggest that PG1 expression in PG1 Tg KC primary cells was not sufficient to induce active migration of the cells. This is consistent with the fact that the expression of PG1 in the skin of PG1 Tg mice is lower than that in other tissues [37]. Furthermore, this suggests that circulating PG1 expressed in other tissues of PG1 Tg mice might be an additional source of PG1 for KCs in the infected wound. In addition, the result of the Boyden chamber assay showed a 20% and 30% increase in the migration of human KCs (HaCaT) and intestinal porcine epithelial cells (IPEC-J2), respectively, after treatment with 4–8 μg/mL of PG1. The basal migration rate of the cells without PG1 treatment was minimal (<3%), which is consistent with the results of the scratch assay. At the highest tested concentration (16 μg/mL) of PG1 treatment, the migration rate was lower than that at 8 μg/mL for both cell lines, which can presumably be attributed to PG1 toxicity [34]. In addition, the migration rate after PG1 treatment was slightly higher in IPEC-J2 cells than that in HaCaT cells, indicating differences in migratory responses depending on the cell type (Figure 5).

### 2.6. Immunomodulatory Effects of PG1 on Epidermal KCs

**Figure 6:**
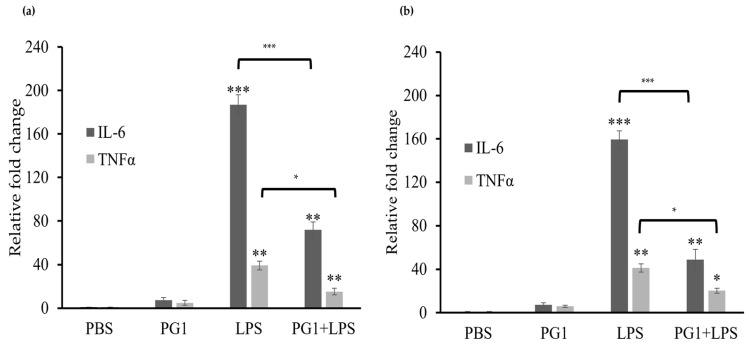
Effect of PG1 treatment on the expression of pro-inflammatory cytokines in primary keratinocytes derived from wild type and PG1 Tg mice in response to lipopolysaccharide (LPS) stimulation. mRNA expression of tumor necrosis factor α and interleukin-6 in primary keratinocytes of (**a**) PG1 Tg and (**b**) wildtype mice following PG1 treatment (10 μg/mL) in the presence or absence of LPS (100 ng/mL). The expression of the target genes was determined using real-time qPCR and was normalized to the expression of *GAPDH* and indicated as relative fold change compared to the unstimulated control group (PBS). All experiments were repeated thrice, and the error bars represent standard deviation. Statistical significance was estimated using unpaired *t*-test; * *p* < 0.01, ** *p* < 0.005, and *** *p* < 0.001. IL, interleukin; LPS, lipopolysaccharide; PBS, phosphate-buffered saline; PG1, protegrin-1; TNF, tumor necrosis factor.

The inflammatory phase is crucial for effective wound healing [1,3]. During this phase, several immune cells, including macrophages and neutrophils, infiltrate the wound tissues and produce pro-inflammatory cytokines [42]. The attenuation of pro-inflammatory cytokines, including interleukin (IL)-6 and tumor necrosis factor alpha (TNFα), promotes cell proliferation and migration during wound healing [42,43]. Therefore, herein, we evaluated the effect of PG1 treatment on the mRNA expression of inflammatory cytokines in PG1 Tg- and wildtype-derived KCs. Cotreatment of PG1 and lipopolysaccharide (LPS) to serve as a pro-inflammatory signal resulted in a significant reduction in the mRNA expression of *IL-6* and *TNFα* (*p* < 0.001) in both PG1 Tg- (Figure 6a) and wildtype-derived KCs (Figure 6b) compared to that in LPS-stimulated cells, suggesting that PG1 exerts immunomodulatory effects in addition to bactericidal effects. However, the levels of pro-inflammatory cytokines after LPS treatment were similar between PG1 Tg- and wildtype-derived KCs, suggesting that the level of PG1 expressed in PG1 Tg KCs is not sufficient to suppress the drastic increase in the expression of pro-inflammatory cytokines in response to LPS treatment.

Mitogen-activated protein kinase (MAPK) signaling pathways reportedly promote wound healing [44,45]. Epidermal growth factor receptor (EGFR) and insulin-like growth factor 1 receptor (IGF1R) are the two most important signaling pathways that are involved in cell proliferation and immunomodulation [15,46,47]. These pathways activate downstream MAPKs to induce cell proliferation and migration. LL37 and several other AMPs induce cell migration by stimulating EGFR expression [13,17,27,48]. We previously showed that HaCaT cell migration can be induced by an increase in EGFR expression following treatment with the python cathelicidin PbCATH4 [13]. Further, PG1 was reported to activate the IGF1R pathway directly or indirectly in IPEC-J2 cells and promote cell migration via EGFR activation [15]. Activation of IGF1R can induce the expression of several innate immune responses, including pro-inflammatory cytokines (IL6 and cyclooxygenase-2) and the migration factor chemokine ligand 2 [15], and these immunomodulatory effects can enhance wound healing. Therefore, the increase in cell migration induced PG1 treatment may be regulated through the same pathway as LL37 and PbCATH4 [13,27], which needs to be investigated in the future.

Wound healing involves several processes, including inflammation, re-epithelialization, and remodeling [6]. Wound infections impair healing and lead to secondary complications and even death [7,8]. Wound infections associated with MDR pathogens, elderly patients with diabetes, and other complications pose significant clinical challenges [4,5,6,7]. Therefore, identifying novel molecules for the topical treatment of infected wounds, especially in cases with other complications, is important. AMPs are promising therapeutic candidates because of their broad-spectrum anti-microbial activity and immunomodulatory effects. Several studies have shown the efficacy of AMPs in wound healing in vitro [13,26,31]. However, studies using in vivo models, such as Tg animals, on the role of AMPs in wound healing and the underlying biological mechanisms are limited. Such studies are needed and will greatly contribute to the understanding of the systematic effects of AMPs during wound healing.

In conclusion, we successfully demonstrated that the ectopic expression of PG1, a pig cathelicidin, in PG1 Tg mice was associated with bacterial clearance and healing in *S. aureus*-infected wounds through re-epithelization without the use of conventional antibiotics. Although the bacterial strains used for the experimental challenge in this study were not clinical MDR pathogens, we expect similar results with MDR pathogens because cathelicidins have been shown to be effective against such strains [49,50,51,52]. In addition, we showed that in vitro treatment of primary KCs with recombinant PG1 stimulated cell migration and proliferation. Our results on the wound-healing effect of PG1, based on a Tg model, confirm that AMPs are promising viable alternatives to existing drugs for wound healing.

## 3. Materials and Methods

### 3.1. Animal Experiments

PG1 Tg mice were generated using preproPG1 under the control of the *MUC1* promoter in a C57BL/6J background, as reported previously [37]. Hence, C57BL/6J mice were used as wild-type mice (Orient Bio Inc., Seongnam, Republic of Korea). Twenty-five (six-week-old) PG1 and wild-type mice were housed individually in specific pathogen-free conditions with unrestricted feeding on a 12 h light–dark cycle and allowed to acclimatize to the environment for one week. 

The hair on the back of the mice was shaved off, and the skin was sterilized with 70% ethanol. Subsequently, an 8 mm full-thickness wound was created aseptically using a disposable biopsy punch (BP-80F, Kai-Medical, Honolulu, HI, USA) under anesthesia using isoflurane (657801261, Hana Pharma, Daejeon, Republic of Korea). Anesthesia was maintained with 1–2% isoflurane using a mouse gas mask and an anesthesia vaporizer (Scitech Korea, Daejeon, Republic of Korea). The wounds were then infected with 40 μL of 10^6^ colony forming units (CFUs) of *S. aureus* ATCC 6538 suspended in 1x phosphate-buffered saline (PBS). 

For antibiotic treatment, 2 μg of gentamycin (345814-1GM, Sigma-Aldrich, St. Louis, MO, USA) dissolved in 40 μL of 1x PBS was applied to the wounds under anesthesia every 24 h for 3 days. The condition of the wounds was examined 3, 7, and 14 days after infection. The wounds were imaged, and the size of the wound area was determined for each animal using ImageJ software version 1.53t [53]. The wound area percentage was calculated based on the proportion of unclosed wounds in each sample relative to the wound size on day 0. The statistical significance of the difference in wound area size between the various treatment groups was determined using Student’s *t*-test. 

For tissue biopsy, the animals were sacrificed via CO_2_ inhalation. Wounds were excised and bisected in the middle, and each portion was used to determine the bacterial count and histological analysis. Five mice were analyzed at each time point. All animal care and experimental protocols were approved and supervised by the Institute of Animal Care and Use Committee of Konkuk University (KU15119).

### 3.2. Determination of Bacteria Load in Wound Biopsies

The wound tissues were weighed and homogenized in 500 μL of 1x PBS for 5 min using a handheld homogenizer (Innogene Tech, Seoul, Republic of Korea). The mixtures were serially diluted 10^7^-fold, and 50 μL of each dilution was plated in Luria Broth agar (LB, 244520, BD Biosciences, Franklin Lakes, NJ, USA) plates. The number of colonies was counted, and the log_10_ CFUs per gram of wound tissue was calculated.

### 3.3. Histological Analyses

Wound biopsies were fixed for 24 h in 4% paraformaldehyde (158127-100G, Sigma-Aldrich) in PBS and processed using a tissue processor (Lecia Biosystems, Wetzlar, Germany). The tissues were embedded in paraffin (39601006, Lecia Biosystems), and 5 μm-thick sections were cut using a microtome (Lecia Biosystems). Sections were then deparaffinized using xylene (534056-500ML, Sigma-Aldrich) and rehydrated using a graded series of ethanol solutions. For hematoxylin and eosin staining, the sections were stained for 5 min with hematoxylin (03971-250ML, Sigma-Aldrich) and counterstained with eosin (318906-500ML, Sigma-Aldrich) for 1 min. For Gram staining, deparaffinized and dehydrated sections were stained with crystal violet and counterstained with safranin using a Gram staining kit (77730-1KT-F, Sigma-Aldrich) according to the manufacturer’s protocol. Images were captured using a BX51 microscope (Olympus, Tokyo, Japan).

### 3.4. KC Isolation

Primary KCs were isolated from the tails of 6-week-old adult PG1 Tg mice, as described previously [54]. Briefly, two mice were sacrificed, and their tails were cut using a surgical blade. A midline cut was generated using a blade to expose the tailbone, which was gently peeled off using forceps. The tail skin was further cut into 2–3 cm pieces and rinsed with 1x sterile PBS. Multiple skin pieces were placed in a 15 mL tube containing 12 mL of dispase (4 mg/mL, D4693-1G, Sigma-Aldrich) digestion mix prepared in Keratinocyte Growth Medium 2 (C-39011, Sigma-Aldrich). The tail tissue was digested overnight at 4 °C via gentle shaking. The digested tissue was rinsed in sterile 1x PBS to remove excess dispase and placed in a sterile Petri dish with the epidermal side of the skin facing the bottom of the dish. The dermal side was incubated with 500–700 μL of trypsin (T-4049, Sigma-Aldrich) for 5 min at room temperature, following which the dermal tissue was removed using forceps, and trypsin was added to the remaining basal layer of the skin and incubated for 30 min at room temperature. The basal layer of the skin was then vigorously rubbed in a Petri dish containing 2–3 mL Keratinocyte Growth Medium 2 (Sigma-Aldrich) to release the cells from the epidermal sheets. This process was further repeated thrice, and the cells were then gently pipetted up and down several times to break up any clumps. The cell suspension was filtered using a 100 μm filter (93100, SPL Lifesciences, Pocheon-si, Republic of Korea) and centrifuged at 180× *g* for 5 min. The primary KCs were counted using a hemacytometer (Z359629, Sigma Aldrich), and 10^5^ cells were plated per well in 6-well plates (30006, SPL Lifesciences) containing Keratinocyte Growth Medium 2 supplemented with 15% fetal bovine serum (FBS) (F2442, Sigma-Aldrich) and cultured in a humidified chamber with 5% CO_2_ at 37 °C.

### 3.5. Production of Recombinant PG1 Peptides

PG1 peptides were produced using a previously reported PG1 expression construct based on a green fluorescent protein (GFP) scaffold system [39]. Briefly, the PG1 construct was transformed into *Escherichia coli* BL21 (DE3) cells (CMC0014, Sigma-Aldrich). PG1 expression was then induced using 0.1 mM Isopropyl β-d-1-thiogalactopyranoside (I6758-5G, Sigma-Aldrich) for 5 h in 1 L of LB medium. The pellets were lysed, and insoluble proteins were extracted. The target protein was purified using a Ni-NTA column (29051021, Cytiva, Marlborough, MA, USA). PG1 was released from the GFP scaffold by cyanogen bromide (CNBr) digestion (C91492, Sigma-Aldrich). The digested mixture was dialyzed against water to remove the CNBr and then lyophilized. PG1 peptides were then separated by reverse-phase high-performance liquid chromatography using a C18 column (WAT011803, Waters, Milford, MA, USA), and the target peptides were analyzed using 16% Tris-tricine SDS-PAGE to estimate purity. The target fractions were lyophilized and stored as a powder at −20 °C until further use.

### 3.6. Determination of PG1 Antimicrobial Activity 

The antimicrobial activities of recombinant PG1 were determined against *S. aureus* ATCC 6538 and *E. coli* ATCC. Microtiter assays were performed in accordance with the guidelines of the Clinical and Laboratory Standards Institute (2018) [55] with minor modifications. Briefly, 4 to 5 single colonies were inoculated and cultured in LB medium for 6 to 8 h. The cells were then washed with sterile saline and resuspended in Mueller–Hinton (MH) broth (275710, BD Biosciences). Bacteria (10^5^ CFUs/well) were seeded in 96-well plates containing 180 μL of MH broth. Various concentrations of PG1 (1 to 10 μg) were added to the wells. Gentamycin and 1x PBS were used as the positive and negative controls, respectively. The plates were incubated for 6 h at 37 °C with shaking. The cell viability count was determined using a Microbial Viability Kit-WST, according to the manufacturer’s protocol (M439, Dojindo, Kumamoto, Japan) and as described previously [56]. Coloring reagent (10 μL) was then added to the wells and incubated for 2 h. The absorbance was read at 450 nm using a spectrophotometer (xMark™ spectrophotometer, Bio-Rad, Hercules, CA, USA). The minimum inhibitory concentration was determined by measuring the optical density of the treated wells and negative controls. All experiments were repeated thrice.

### 3.7. Cell Scratch Assay

PG1 Tg KCs were grown to 90% to 95% confluency in Keratinocyte Growth Medium 2 (Sigma-Aldrich) supplemented with 15% FBS (Sigma-Aldrich), and then serum starved for 12 h. The plates were scratched with a 200 μL micropipette tip and washed thrice with 1× PBS to remove detached cells. Fresh culture medium supplemented with 5% FBS was added to the remaining attached cells either with or without recombinant PG1 (8 μg/mL). Changes in the scratched areas were monitored by taking images at the same location at 0, 12, and 24 h using a Nikon T2 Eclipse microscope (Nikon, Tokyo, Japan).

### 3.8. Migration Assay

Migration assay was performed using an 8 μM insert in a Boyden chamber (36224, Transwell® chamber, SPL Life Science, Pocheon-si, Republic of Korea) using HaCaT (CLS, Eppelheim, Germany) and IPEC-J2 (DSMZ-German collection of Microorganism and Cell cultures, Brunswick, Germany) cells. Briefly, 1.0 × 10^4^ cells were seeded into the upper chamber containing Dulbecco’s modified Eagle’s medium (DMEM; D5796, Sigma-Aldrich) supplemented with 10% inactivated FBS. PG1 (2, 4, 8, and 16 μg/mL) was added to the bottom chamber containing DMEM with 10% inactivated FBS, and 1x PBS was used as the negative control. Cells were incubated at 37 °C with 5% CO_2_ for 16 h. The outer side of the insert membrane was precoated with 0.5% gelatin (G9391, Sigma-Aldrich). The cells that adhered to the upper chamber were removed using cotton buds, and the cells that migrated through the membrane and adhered to the lower side were stained using Giemsa stain (G4507, Sigma-Aldrich). The membranes were carefully mounted on a glass slide, and the number of migrated cells was counted in five random microscopic fields at 400× magnification. All experiments were repeated thrice.

### 3.9. Real-Time Quantitative PCR

Primary KCs from both wildtype (C57BL/6) and PG1 Tg mice were treated with 10 μg/mL of recombinant purified PG1 in the presence or absence of *E. coli* O111:B4 LPS (100 ng/mL) (LPS25, Sigma Aldrich) for 12 h. Total RNA was extracted using the Qiagen RNA extraction kit (74104, Qiagen, Germantown, MD, USA) according to the manufacturer’s protocol. cDNA was synthesized using Superscript III reverse transcriptase kit (RT006L, Enzynomics, Daejeon, Republic of Korea) according to the manufacturer’s protocol. Real-time quantitative PCR was performed using the SYBR-Green supermix (1725270, Bio-Rad) using CFX connect Real-Time PCR system (Bio-Rad). The following thermocycling conditions were used: 95 °C for 2 min, followed by 40 cycles of 95 °C for 15 s and 59 °C for 30 s. The primers used in the present study are listed in Table S2. Target gene expression was expressed as relative fold change by comparing with the expression of the housekeeping gene (mice *GADPH*). Relative gene expression was calculated using the 2-ΔΔCT method [57].

## Figures and Tables

**Table 1:** Temporal changes in the bacterial load in wounds of PG1 transgenic mice.

Experimental Conditions	Day 0 (CFUs/g)	Day 3 (CFUs/g)	Day 7 (CFUs/g)	Day 14 (CFUs/g)
PG1 mice with uninfected wounds (group 1) ^1^	0	0	3 ± 4.3	5 ± 5.1
Wild-type mice with *S. aureus*-infected wounds (group 2) ^1^	1.85 ± 0.8 × 10^6^ *	8.21 ± 3.4 × 10^8^	8.75 ± 3.2 × 10^8^	Fatal
Wild-type mice with *S. aureus*-infected wounds treated with gentamycin for 3 days (group 3) ^1^	1.85 ± 0.8 × 10^6^ *	4.89 ± 2.3 × 10^5^	1.41 ± 0.9 × 10^2^	41 ± 3.8
PG1 transgenic mouse with *S. aureus*-infected wounds that were not subjected to antibiotic treatment (group 4) ^1^	1.85 ± 0.8 × 10^6^ *	6.5 ± 2.7 × 10^4^	1.39 ± 2.4 × 10^2^	31 ± 4.2

^1^ Five mice were analyzed at each time point. CFUs, colony-forming units; 1 × 10^6^ CFUs of *S. aureus* were inoculated for infection. Fatal, none of the mice survived. * The average CFU value on day 0.

## Data Availability

Data are contained within the article or Supplementary Material.

## Supplementary Materials

The following supporting information can be downloaded at: https://www.mdpi.com/article/10.3390/ijms241411658/s1.
