# Supplementary material for: Effective Healing of Staphylococcus aureus-Infected Wounds in Pig Cathelicidin Protegrin-1-Overexpressing Transgenic Mice
Source: Int J Mol Sci. 2023 Jul 19;24(14):11658. doi: 10.3390/ijms241411658 (PMC10380341; doi:10.3390/ijms241411658)
Supplement: Supplementary file 1 [file ijms-24-11658-s001.zip › ijms-2385012-supplementary.pdf]

International Journal of *Molecular Sciences*

MDPI

Supplementary Materials

Effective Healing of *Staphylococcus aureus*-Infected Wounds in Pig Cathelicidin Protegrin-1-Overexpressing Transgenic Mice

Nagasundarapandian Soundrarajan, Prathap Somasundaram, Dohun Kim, Hye-Sun Cho, Hyoim Jeon, Byeonyong Ahn, Mingue Kang, Hyuk Song and Chankyu Park *

Department of Stem Cells and Regenerative Biology, Konkuk University, Hwayang-dong, Seoul 05029, Republic of Korea; sundarmeets@gmail.com (N.S.); prathaparunms@gmail.com (P.S.); kdh2136@konkuk.ac.kr (D.K.); chssky77@gmail.com (H.-S.C.); kamuijhi@naver.com (H.J.); anhbyeongyong1123@gmail.com (B.A.); mingue5349@gmail.com (M.K.); songh@konkuk.ac.kr (H.S.)

* Correspondence: chankyu@konkuk.ac.kr; Tel.: +82-2-450-3697; Fax: +82-2-450-0686

Table S1. Evaluation of minimum inhibitory concentration of recombinant PG1.

Strains	PG1 (μg/mL)	Gentamycin (μg/mL)
Staphylococcus aureus ATCC 6538	4	2
Escherichia coli ATCC 25922	6	1

Table S2. Primers used in the Real-time quantitative PCR.

Name	Primer Sequence (5' to 3')	Species Amplified
IL-6-F	TACCACTTCACAAGTCGGAGGC	Mouse
IL-6-R	CTGCAAGTGCATCATCGTTGTTC	Mouse
TNFα-F	GCCTCTTCTCATTCCTGCTTG	Mouse
TNFα-R	CTGATGAGAGGGAGGCCATT	Mouse
GAPDH-F	GTGAAGGTCGGTGTGAACGGATT	Mouse
GAPDH-R	GGAGATGATGACCCTTTTGGCTC	Mouse

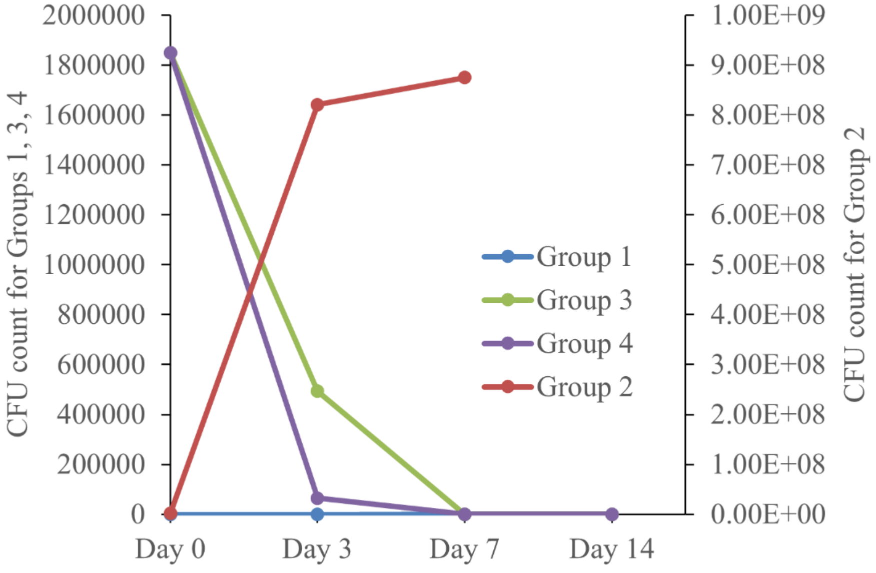

Figure S1. Estimation of bacterial loads in wound biopsy. Five mice per group were used to estimate the colony forming unit (CFU)/gram wound biopsy tissue. Group 1; PG1 Tg mice without infection, Group 2; wildtype mice with infection, Group 3; wildtype mice with infection and treated with gentamycin, and Group 4; PG1 Tg mice with infection. Both Group 3 and 4 showed significant

reduction of bacterial count on day 7 and 14. Group 2 to 4 were infected with 1.0 × 10^6 CFU of *S. aureus*.

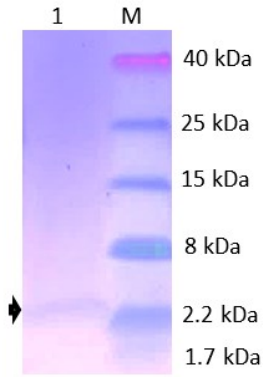

Figure S2. Recombinant PG1 purified by RP-HPLC. Lane 1: Recombinant PG1 purified by RP-HPLC analyzed by 16% Tris-tricine SDS-PAGE. The target band corresponding to PG1 is indicated by an arrow. Lane M, low-molecular-weight marker (Lane M). The PG1 purity was > 95% according to SDS-PAGE.
